# Are Dentists Honest?

**Published:** 1890-11

**Authors:** 


### Are Dentists Honest?

Educated people who care for their teeth in a proper manner select some dentist whom they believe to be competent and honest; competent meaning skillful, well posted; honest the same as any respectable business directs. It does happen, however, that some will fall into the hands of a pretentious charlatan who, by slippery ways manage to collect large fees for a time, and then his tricks being discovered, drops the patient because the patient drops him. Then comes the rub. The patient at last discovers that irretrievable damage has been done upon his once fine set of teeth, and in this dubious hour imprecations are heaped upon the whole dental fraternity. Friends of the victim take up the cry and it instills suspicion of every dentist. Fortunately, we as a profession, are not alone in this manner of persecution; other, and in fact all professions have their admirers and their enemies. Dentists are, as a rule, compelled to be honest to their patients from the fact that the dental nerves are so extremely sensitive that improper procedures result in pain necessitating *a priori*, the best operative procedures that will insure immunity from pain and a continued arrest of disease.

That there are dishonest dentists we do not doubt. Attention has recently been directed to a class of dentists who prey upon the credulity of the public by exhibition of a State directory containing their names with the name of the college from which they graduated (?). In an instance we know of a puddler from an iron mill takes up the forceps, hangs out his shingle, and has his name placed in a directory of the Dentists of Ohio, and goes to puddling around in pulp canals and traversing gory fields, and directs attention of the public to the fact that he is a graduate of a respectable dental college. This and other false representations calls forth the expression "Where are the dental laws of Ohio?"

But it may be said with pride that the dental profession, as a whole, in the State of Ohio is composed of honest laborers in their chosen field, and none regret the stupidity and bad conduct of some more than they. We hope the dental law of Ohio will be amended to remedy many evils. W.

---

Dental Surgery for Medical Practitioners and Students of Medicine, by A. W. Barrett, M. B., London, and M.R.C.S., L.D.S.E., Dental Surgeon of the London Hospital; Lecturer on Dental Surgery in the Medical School. Second edition with illustrations.

This little work is from the press of P. Blakeston, Son & Co., Philadelphia. It is a very concisely written, and condensed presentation of the subjects upon which it treats, such as are interesting to, and ought to be understood by all medical students and medical practitioners. To them it will be of great value. It should be in the hands of, and studied by, every medical student. The information condensed into one hundred and thirty-six pages in this book can not be found in the ordinary medical text-books without very great labor. It would also be of value to the dental student as well; but all the matters presented here will be found in the ordinary text-books of the dental course. But the presentation in this little work is very good indeed, and may with great profit be examined by the dental student and dental practitioner.

